# Chloride electrode composed of ubiquitous elements for high-energy-density all-solid-state sodium-ion batteries

**DOI:** 10.1038/s41598-024-53154-5

**Published:** 2024-02-01

**Authors:** Naoto Tanibata, Naoki Nonaka, Keisuke Makino, Hayami Takeda, Masanobu Nakayama

**Affiliations:** Department of Advanced Ceramics, Nagoya Institute of Technology, Gokiso, Showa, Nagoya, Aichi 466-8555 Japan

**Keywords:** Materials for energy and catalysis, Batteries, Batteries

## Abstract

Inexpensive and safe energy-storage batteries with high energy densities are in high demand (e.g., for electric vehicles and grid-level renewable energy storage). This study focused on using NaFeCl_4_, comprising ubiquitous elements, as an electrode material for all-solid-state sodium-ion batteries. Monoclinic NaFeCl_4_, expected to be the most resource-attractive Fe redox material, is also thermodynamically stable. The Fe^2+/3+^ redox reaction of the monoclinic NaFeCl_4_ electrode has a higher potential (3.45 V vs. Na/Na^+^) than conventional oxide electrodes (e.g., Fe_2_O_3_ with 1.5 V vs. Na/Na^+^) because of the noble properties of chlorine. Additionally, NaFeCl_4_ exhibits unusually high deformability (99% of the relative density of the pellet) upon uniaxial pressing (382 MPa) at 298 K. NaFeCl_4_ operates at 333 K in an electrode system containing no electrolyte, thereby realizing next-generation all-solid-state batteries with high safety. A high energy density per positive electrode of 281 Wh kg^−1^ was achieved using only a simple powder press.

## Introduction

The demand for energy-storage batteries to realize a sustainable society is increasing annually^1^. Lithium-ion batteries are used in various portable devices because of their high working potentials (> 3 V)^2^. However, the low abundance of lithium and redox-center transition metals (such as Co and Ni)^3,4^ and their environmental impact, such as the soil contamination associated with their extraction, have become problematic^5^. Alternative solutions that have been proposed for these problems include the use of sodium-ion batteries, which are composed of ubiquitous elements, including sodium as the carrier ion, which has chemical properties similar to those of lithium, such as a high ionization tendency and stable monovalent ions^6,7^. In addition, commercially available lithium-ion batteries currently use flammable organic electrolytes, and their safety is a concern as their application in electric vehicles and large storage batteries continues to advance. In view of these concerns, all-solid-state batteries (ASSBs), which have inorganic solid electrolytes, are expected to be used as next-generation batteries because of their highly improved safety^8–10^. Furthermore, because ASSBs do not contain liquid electrolytes, new electrode materials like elemental sulfur and chloride, which would otherwise leach into the liquid electrolyte, can be used, thereby creating the possibility of increasing the energy density beyond those of conventional batteries^11,12^.

Chlorides are being extensively studied as solid electrolytes for ASSBs^13,14^. Compared to compounds with divalent anions, such as oxides and sulfides, which have received widespread attention, anionic compounds with a univalent anion, such as chlorides, undergo limited Coulombic interactions with carrier ions, which have high diffusivity^15^. Unlike oxides, chloride ions tend to exhibit high polarizability and deformability, similar to that of sulfides^16–18^. High deformability enables the use of a sinterless compaction process, which is advantageous for the fabrication of ASSBs^19^. The disadvantage of the use of sintering for densification is that it is inapplicable to certain materials owing to side reactions^20^ and the evaporation of elements^21,22^.

An additional benefit of chloride is that it is a promising electrolyte, particularly in cathodes, owing to its higher oxidation resistance (~ 4.0 V vs. Li/Li^+^) than those of sulfide (~ 2.5 V vs. Li/Li^+^) and oxide (~ 3.5 V vs. Li/Li^+^), which has its origins in the noble properties of chlorine^23,24^. This high resistance to oxidation is a feature of the high working potential required for high energy density when used in electrode materials. As mentioned previously, the high polarity of chloride electrolytes often causes dissolution of the constituent elements in the electrolyte solution when they are used as liquid electrolytes. This severely affects the cycle life of the battery because it lowers the number of reversible charge–discharge cycles. However, the dissolution reaction of chloride electrodes is suppressed when they are used in combination with solid electrolytes; thus, chloride electrodes are potentially suitable for use as high-voltage cathode materials for rechargeable ASSBs.

In this study, we focused on NaFeCl_4_ as an electrode material for ASSBs, because NaFeCl_4_ contains sodium chloride and the ubiquitous element Fe as the transition metal in the redox center. NaFeCl_4_ is registered on the ISCD (#16994)^25^ and has an orthorhombic crystal structure (*S.G.*: *P2*_*1*_*2*_*1*_*2*_*1*_), with Fe in the center of the tetrahedron formed by the chloride ions. In electrode materials based on the Fe^2+/3+^ redox reaction, Na_2_Fe_2_(SO_4_)_3_^26^ exhibited a higher potential (3.8 V vs. Na/Na^+^) than conventional oxide electrodes (Fe_2_O_3_^27^, 1.5 V vs. Na/Na^+^) because of the noble properties of the SO_4_^2−^ unit (which has an inductive effect). The same effect that was induced by the SO_4_^2−^ ion was expected for the lighter Cl^−^ ion (molar mass per charge Cl^−^: 34.5 g mol^−1^, (SO_4_^2−^)/2:48 g mol^−1^), and the charge–discharge properties of the Fe^2+/3+^ redox pair were evaluated at the NaFeCl_4_ electrode. The theoretical capacity of this material is 121 mAh g^−1^ at one Na per NaFeCl_4_ formula unit. We also assembled an ASSB using an electrode without electrolyte added to the electrode composite (hereafter referred to as an “electrolyte-free electrode”). Conventional oxide electrodes exhibit low deformability and can be fabricated by adding soft sulfide electrolytes or other materials. However, the addition of electrolytes has the effect of decreasing the theoretical energy density per electrode composite, in which case the reaction distribution becomes more complicated. For chloride electrodes, the active material in the electrode may have high deformability. Therefore, in ASSBs, the electrode composites that do not contain solid electrolyte powders may surpass conventional battery systems in terms of their energy density.

## Results and discussion

### Structure and deformability

**Figure 1:**
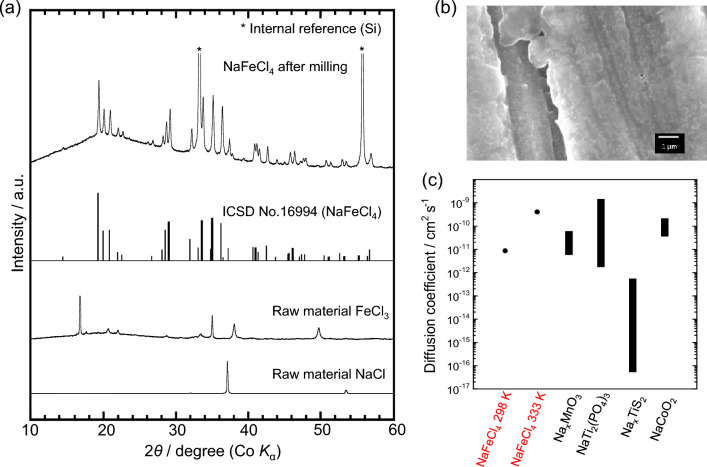
Characterization of synthesized NaFeCl_4_. (**a**) XRD patterns of NaFeCl_4_ after milling and the raw material and the simulated pattern of orthorhombic NaFeCl_4_ from the ICSD database (#16994). (**b**) Cross-sectional SEM image of the pellet uniaxially compressed. (**c**) Conductivity diffusion coefficient of NaFeCl_4_ obtained from AC impedance measurements at 298 and 333 K and the chemical diffusion coefficients of typical oxide (Na_x_MnO_3_^30^,NaTi_2_(PO_4_)_3_^31^,and NaCoO_2_^32^) and sulfide (Na_x_TiS_2_^33^) cathode materials at room temperature.

The X-ray diffraction (XRD) pattern of the synthesized NaFeCl_4_ sample (Fig. 1a) indicates a single phase consisting of monoclinic NaFeCl_4_. The peaks of the raw material are no longer visible. The relative density of the uniaxially compressed pellet was calculated from the apparent density of the compact and crystal lattice density of the monoclinic NaFeCl_4_ (2.31 g cm^−3^). The value of 99.1% for NaFeCl_4_ (at 382 MPa) is higher than those for Li_2_FeCl_4_ (92% at 382 MPa)^28^ and Li_3_TiCl_6_ (86.1% at 350 MPa)^29^, which have recently been reported as highly deformable electrodes. The cross-sectional scanning electron microscopy (SEM) image of the NaFeCl_4_ powder compact (Fig. 1b) indicates that the grains were crushed by compaction, resulting in a dense structure with ill-defined grain boundaries. These results indicate that the NaFeCl_4_ powder has high deformability.

### Electrochemical performance

**Figure 2:**
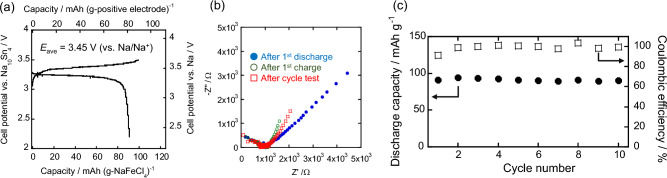
Characteristics of the ASSB (Na_10_Sn_4_ + AB|Na_3_PS_4_|Na_2.25_Y_0.25_Zr_0.75_Cl_6_|NaFeCl_4_ + KB) using a NaFeCl_4_ electrode without electrolyte (NaFeCl_4_:KB = 90:10 wt%) and with the bilayer electrolyte (Na_3_PS_4_|Na_2.25_Y_0.25_Zr_0.75_Cl_6_). (**a**) Constant-current charge–discharge curves. (**b**) Impedance plots before and after charging and discharging. (**c**) Cycle characteristics of discharge capacity (circle) and Coulombic efficiency (square).

An ASSB with a solid electrolyte was fabricated to evaluate this strongly ionic electrode-active material without it leaching into the electrolyte. In addition, taking advantage of this deformability, electrolyte-free electrodes were fabricated for application in ASSBs, and their charge–discharge characteristics were evaluated. Based on the conductivity diffusion coefficient (Fig. 1c) obtained from the impedance plot (Supplementary Fig. 1), the battery operating temperature was set at 333 K to ensure that the diffusion coefficient is higher than those of conventional electrode materials. The solid electrolyte (Na_3_PS_4_|Na_2.25_Y_0.25_Zr_0.75_Cl_6_) consists of two layers, one on the anode (Na_10_Sn_4_) side and the other on the cathode (NaFeCl_4_) side, to suppress the reactions between the electrodes and electrolytes (cell configuration: Na_10_Sn_4_ + acetylene black (AB)|Na_3_PS_4_|Na_2.25_Y_0.25_Zr_0.75_Cl_6_|NaFeCl_4_ + KB) based on an examination of reactions like the oxidation of Na_3_PS_4_, as shown in Supporting Section 1 (Supplementary Figs. S2 and S3). The resulting reversible capacity was 90.8 mAh (g-NaFeCl_4_)^−1^ (81.7 mAh (g-positive electrode)^−1^), and the average working potential was ~ 3.45 V (vs. Na/Na^+^), as is evident from the constant-current charge–discharge curves (Fig. 2a) and the d*Q*/d*V* curves (Figure S4).

The results of the impedance measurements (Fig. 2b) indicate only a small semicircular resistance and no significant increase in the first charge–discharge process or after the cycle test. The battery also exhibits relatively stable cycling characteristics over 10 cycles (Fig. 2c). Based on the reversible capacity of this discharge capacity (~ 90 mAh g^−1^), the gravimetric energy density per positive electrode was calculated to be 281 Wh kg^−1^ at the reference potential of Na (~ 3.45 V). In Table 1, this value is compared with previously reported energy densities of bulk ASSBs with high-potential operation (> 3 V). This shows that the ASSB fabricated in this study via a simple process using only pressed powders, which does not require any coating or sintering process on the surface of the cathode active material, has higher energy density than other reported bulk ASSBs using inorganic and/or polymer electrolytes.

**Table 1:** Comparison of the energy density per positive electrode weight, *E*_m_^*a*^, of the ASSB in this study with that of other bulk ASSB with a high-potential cathode (> 3 V).

Report	*E*_m_ (Wh kg^−1^)	Cathode active material (CAM)	Type of solid electrolyte	Solid electrolyte (SE)	Weight ratio of cathode (CAM:SE:others)	Preparation	Operating temperature (K)
This experimental	281	NaFeCl_4_	Inorganic	Na_3_PS_4_ and Na_2.25_Y_0.25_Zr_0.75_Cl_6_	90:0:10	Power compress	333
Zhang et al.^34^	278	Na_3_V_2_P_3_O_12_		La-substituted Na_3_Zr_2_Si_2_PO_12_	65:15:20	Sintering	353
Duchêne et al.^35^	185	NaCrO_2_		Na_4_(B_12_H_12_)(B_10_H_10_)	70:20:10	Power compress	303
Yamauchi et al.^36^	173	Na_2_O-Fe_2_O_3_-P_2_O_5_ glass		β”—Al_2_O_3_	72:25:3	Sintering	303
Hayashi et al.^37^	91.6	NaCrO_2_		Na_3_PS_4_	36:55:9	Power compress	303
Niu et al.^38^	169	C_2_H_4_(CN)_2_-NaClO_4_ coated Na_0.67_Ni_0.33_Mn_0.67_O_2_	Polymer & Inorganic	Polyethylene Oxide-Na_3_Zr_2_Si_2_PO_12_-NaClO_4_	70:0:30	Power compress	328
Yao et al.^39^	288	Na_3_V_2_P_3_O_12_	Polymer	Polyethylene glycol dimethacrylate -NaFSI	70:20:10	Power compress	333
Yu et al.^40^	237	Na_2_MnFe(CN)_6_		Polyethylene Oxide-NaClO_4_-Na_3_Zr_2_Si_2_PO_12_	55:18:27	Power compress	333
Zhao et al.^41^	220	Na_3_V_2_P_3_O_12_		Polyethylene Oxide-NaFSI	60:10:30	Power compress	353

^*a*^*E*_m_ is the energy density per positive electrode weight assuming a Na metal anode. If not stated otherwise, it was calculated from the initial discharge curve. Other information, such as the materials used, their mixing ratios, fabrication conditions, and working temperatures, is also shown.

**Figure 3:**
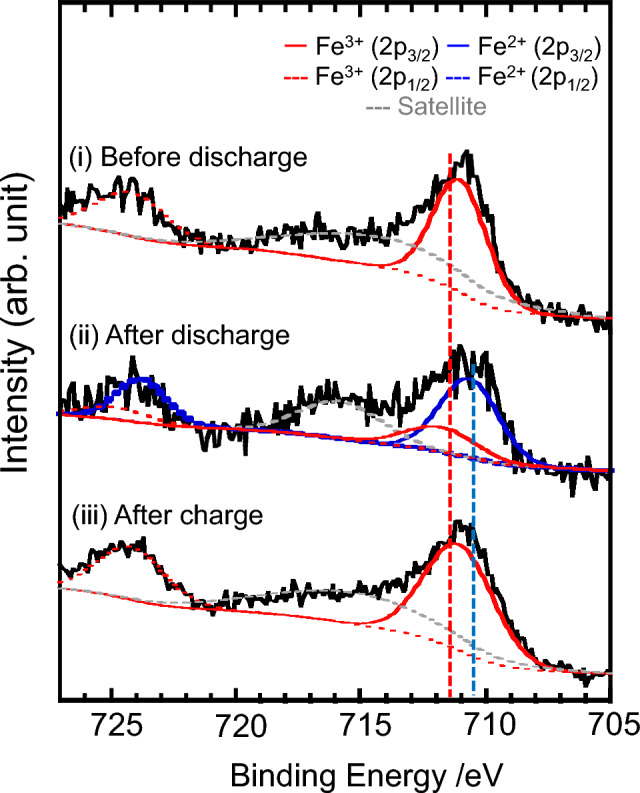
Fe 2p XPS results of the NaFeCl_4_ electrode before and after the charge–discharge process. The spectra were fitted with 2p_3/2_ and 2p_1/2_ of Fe^2+/3+^ and satellite peaks.

The redox mechanism of the NaFeCl_4_ electrode was investigated by X-ray photoelectron spectroscopy (XPS) before and after the charge–discharge process. The Fe 2p XPS profile (Fig. 3) consists of two sets of doublet peaks (Fe 2p_3/2_ and Fe 2p_1/2_) and their satellite peaks. Deconvolution of each spectrum using the pseudo-Voigt function revealed that the Fe 2p_2/3_ peak is located near 711.0 eV before and after charge, whereas a high-intensity peak appears near 710.5 eV, and the intensity of the peak at 711.0 eV is lower after discharge. The Fe 2p_2/3_ peaks of FeCl_2_ and FeCl_3_ in the reference sample appear at 710.6 eV and 711.3 eV, respectively^42,43^, with the low- and high-energy peaks attributable to Fe^2+^ and Fe^3+^, respectively. The peak ratio after discharge was approximately 3:1, which is consistent with the fact that the discharge capacity was approximately 75% of the theoretical capacity (121.5 mAh g^−1^). This indicates that the charge–discharge process proceeded via the redox reaction of Fe^2+/3+^ in NaFeCl_4_. As mentioned previously, the redox reaction of Fe^2+/3+^ has been reported to have a low potential of approximately 1.5 V in conventional oxides (Fe_2_O_3_). In this material, the inductive effect of chlorine may be responsible for the higher potential (3.45 V vs. Na/Na^+^), which would be responsible for the high energy density listed in Table 1. The XPS profile after charging revealed a reversible return to the original Fe^3+^ state before the charge–discharge process. The XRD patterns before and after charging (Supplementary Fig. S4) also show a reversion to monoclinic NaFeCl_4_, indicating the occurrence of a reversible charge–discharge reaction involving a Fe^2+/3+^ redox reaction. The synthesis of Na_2_FeCl_4_, which can be charge-started, has not yet been reported; therefore, it is expected to be evaluated in future studies.

In summary, the NaFeCl_4_ electrode, composed of ubiquitous elements, was evaluated for application in a low-cost storage battery with high energy density and safety. An ASSB was operated at 333 K with an electrolyte-free electrode owing to the high deformability derived from chloride ions (relative density = 99% of the pellet uniaxially compressed at 298 K). In addition, owing to the inductive effect of chloride, high-potential operation (3.45 V vs. Na/Na^+^) was demonstrated with the most attractive Fe redox reaction (Fe^2+/3+^) in terms of the elemental strategy. Consequently, an outstanding energy density (281 Wh (kg-positive electrode)^−1^) was achieved for conventional bulk all-solid-state sodium-ion batteries without sintering or electrode coating treatment. This study demonstrates the potential of NaCl-based materials as high-energy-density electrode materials, which have previously been difficult to evaluate because of their elution into the electrolyte.

## Methods

### Preparation and evaluation of all-solid-state cells using NaFeCl_4_ electrodes

NaFeCl_4_ was synthesized from NaCl (Wako Pure Chemical Industries, Ltd., 99.5%) and FeCl_3_ (Sigma-Aldrich Japan LLC, 99.9%) powders by a mechanochemical method^28^. A stoichiometric mixture was placed in a 45 mL stainless steel pot with 74 ZrO_2_ balls (diameter = 5 mm) and milled using a planetary ball mill apparatus (Fritsch Japan Co., Ltd., P-7 classic-line, Japan) at a rotation speed of 300 rpm for 5 h. The as-produced yellow sample was probed by XRD (MiniFlex 600, Rigaku, Japan, Co*K*α line). The conductivity diffusion coefficient was measured at 298 and 333 K using the AC impedance method (VSP Potentiostat, BioLogic, France) with an AC voltage of 300 mV and a measurement frequency range of 10^2^–10^6^ Hz. Pellets (diameter = 10 mm, thickness =  ~ 0.50 mm) were prepared by sandwiching the powder between stainless steel plates and compressing under 382 MPa at 298 K. The pellets contain 10 wt% of Ketjen black (KB) as a conductivity aid. The cross-section of a pellet was polished with a #2000 file, and cross-sectional images were acquired using SEM (JSM-6360LV, JEOL, Japan) at a voltage of 10 kV. All the procedures were performed under dry Ar gas.

Electrolyte-free electrodes were prepared with NaFeCl_4_, and the charge–discharge characteristics of the all-solid-state sodium-ion batteries were evaluated. The NaFeCl_4_ electrode was mixed with KB (mixing ratio of NaFeCl_4_:KB = 90:10 wt%) as a conductive aid via ball milling at 300 rpm for 1 h. The solid electrolyte was the sulfide Na_3_PS_4_^44^ and/or chloride Na_2.25_Y_0.25_Zr_0.75_Cl_6_^45^, and the negative electrode (counter electrode) was Na_10_Sn_4_-AB (AB: acetylene black; mixing ratio of Na_10_Sn_4_:AB = 90:10 wt%)^46^. For cell fabrication, approximately 60 mg of the electrolyte was first placed in a polycarbonate pressure vessel with a cylindrical inner diameter of 10 mm, sandwiched between two pieces of stainless steel, and pressurized to 96 MPa. Subsequently, the cathode material was placed on one side and pressed at 96 MPa, whereas the anode material was placed on the other side and pressed at 382 MPa. The cells were then screwed in from the top and bottom and restrained. When two layers of the electrolyte were used, approximately 30 mg of the Na_3_PS_4_ electrolyte was used on the anode side and approximately 30 mg of the Na_2.25_Y_0.25_Zr_0.75_Cl_6_ electrolyte on the cathode side. Charging and discharging were evaluated using a potentiostat/galvanostat (VSP Potentiostat, BioLogic, France) at a temperature of 333 K, current density of 6.4 μA cm^−2^, and starting from the discharging process (Na storage). AC impedance measurements were performed in the frequency range of 1 − 10^6^ Hz after charging and discharging. The charge–discharge mechanism of NaFeCl_4_ was investigated by conducting Fe 2p XPS measurements on the electrode before and after charging and discharging using a Cr *K*_α_ radiation source (PHI Quantes, ULVAC-PHI, Inc., USA) without surface etching treatment, which would be a concern in terms of alteration.

### Supplementary Information

Supplementary Information.

## Data Availability

All relevant data are available from the corresponding author on reasonable request.
