# Supplementary material for: Chloride electrode composed of ubiquitous elements for high-energy-density all-solid-state sodium-ion batteries
Source: Sci Rep. 2024 Feb 1;14:2703. doi: 10.1038/s41598-024-53154-5 (PMC10834940; doi:10.1038/s41598-024-53154-5)
Supplement: Supplementary file 1 — Supplementary Information. [file 41598_2024_53154_MOESM1_ESM.docx]

**Supplementary Material for**

**Chloride Electrode Composed of Ubiquitous Elements for High-Energy-Density All-Solid-State Sodium-Ion Batteries**

Naoto Tanibata,* Naoki Nonaka, Keisuke Makino, Hayami Takeda, and Masanobu Nakayama

Department of Advanced Ceramics, Nagoya Institute of Technology, Gokiso, Showa, Nagoya, Aichi 466-8555, Japan

*Correspondence should be addressed to tanibata.naoto@nitech.ac.jp

**
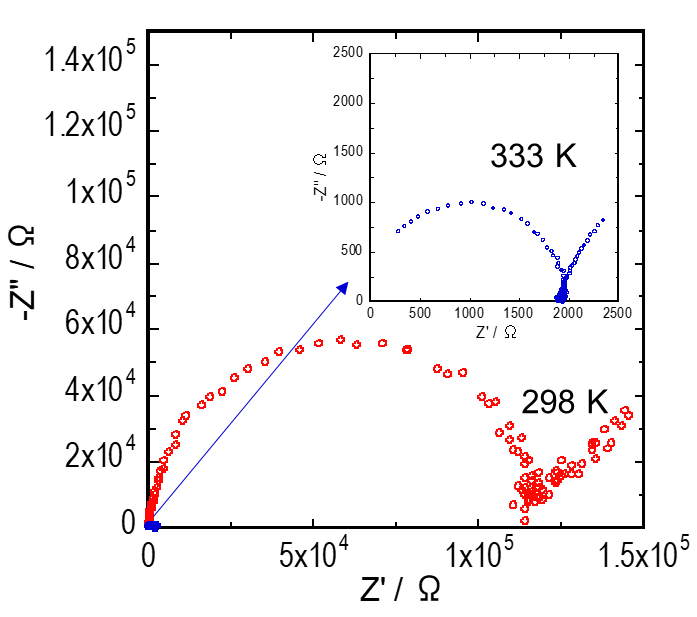
Supplementary Material 1.** **Ionic conductivity of NaFeCl_4_**

**Figure S1.** AC impedance plots of compressed NaFeCl_4_ pellet recorded at 298 K and 333 K.

**Supplementary Material 2. Electrolyte Considerations in All-Solid-State Batteries Evaluating NaFeCl_4_ Electrodes**

Figure S2 shows the results of characterizing the NaFeCl_4_ electrode using the Na_3_PS_4_ sulfide electrolyte, which is the most commonly used solid electrolyte in all-solid-state batteries (ASSBs). The charge–discharge curve (Figure S2a) reveals a low capacity (~30% of the theoretical capacity of 121.5 mAh g^-1^), although it operated at a high average working potential of ~3.45 V (vs. Na/Na^+^). The impedance plot after charging and discharging (Figure S2b) shows two semicircles. The resistance *R*1 on the high-frequency side is that of Na_10_Sn_4_/Na_3_PS_4_ obtained from the results of the Na_10_Sn_4_/Na_3_PS_4_ symmetric cell, while the resistance *R*2 on the low-frequency side is that of the Na_3_PS_4_/NaFeCl_4_ interface because the capacitance is 8.3 × 10^-6^ F after discharge and 1.6 × 10^-6^ F after charge. The resistance is larger than that of Na_10_Sn_4_/Na_3_PS_4_, particularly after charging, showing a significant increase. This may be attributed to the poor oxidation resistance of Na_3_PS_4_ sulfide. Thermodynamic reactivity, checked by the *Interface Reaction App* implemented in the *Materials Project*, also showed that the following reaction proceeds spontaneously (heat of reaction = −1.2 kJ/mol).

Na_3_PS_4_ + 0.9 NaFeCl_4_ → 0.025 P_4_S_9_ + 1.2 NaCl_3_ + 0.175 S + 0.9 Fe

Therefore, we fabricated and evaluated an ASSB using Na_2.25_Y_0.25_Zr_0.75_Cl_6_ as a separator which reportedly exhibits the highest ionic conductivity in an oxidation-resistant chloride electrolyte.

**
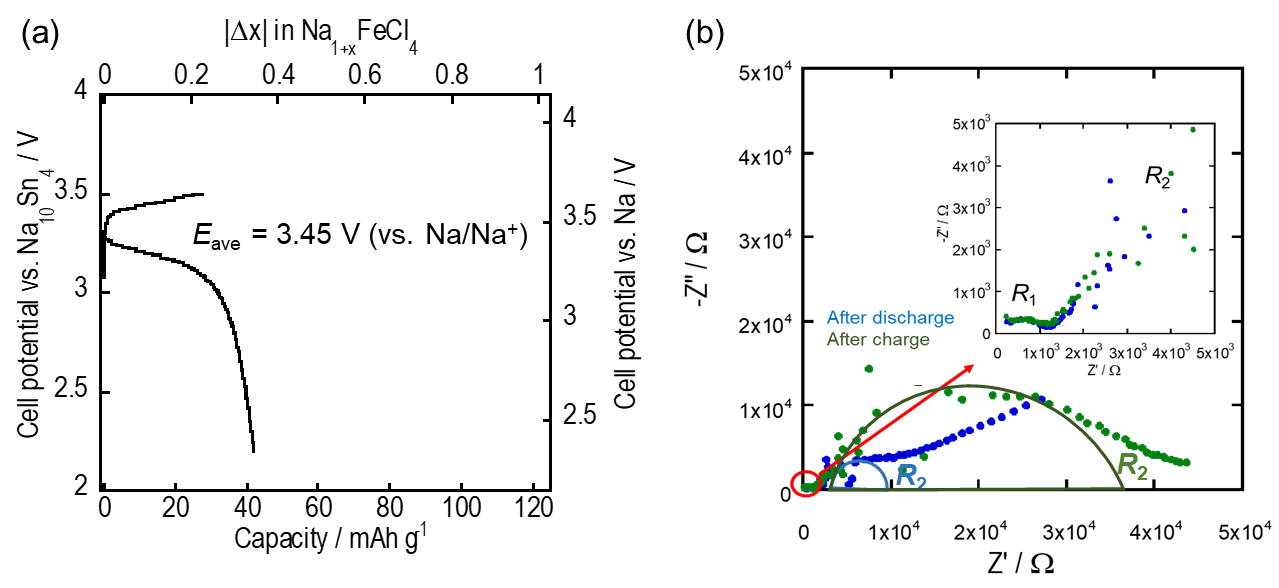
Figure S2.** Characteristics of all-solid-state sodium-ion batteries (Na_10_Sn_4_+AB | Na_3_PS_4_ | NaFeCl_4_+KB) using a NaFeCl_4_ electrode without an electrolyte (NaFeCl_4_:KB = 90:10 wt.%) and with Na_3_PS_4_ sulfide solid electrolyte. (a) Constant-current charge–discharge curves. (b) Impedance plots before and after the charge–discharge process.

In the charge–discharge curve (Figure S3), the average working potential was approximately 0.3 V lower, i.e., 3.2 V (vs. Na/Na^+^) when only Na_2.25_Y_0.25_Zr_0.75_Cl_6_ was used as the separator. The Na_2.25_Y_0.25_Zr_0.75_Cl_6_ electrolyte is known to increase the resistance with each cycle, even when Na–Sn alloys are used. These results suggest that the Na_2.25_Y_0.25_Zr_0.75_Cl_6_ electrolyte is less resistant to reduction than Na_3_PS_4_ and reacts with Na_10_Sn_4_. Therefore, in this study, we used an electrolyte bilayer all-solid-state cell with Na_3_PS_4_ as the electrolyte on the Na_10_Sn_4_ anode side and Na_2.25_Y_0.25_Zr_0.75_Cl_6_ as the electrolyte on the NaFeCl_4_ cathode side.

**
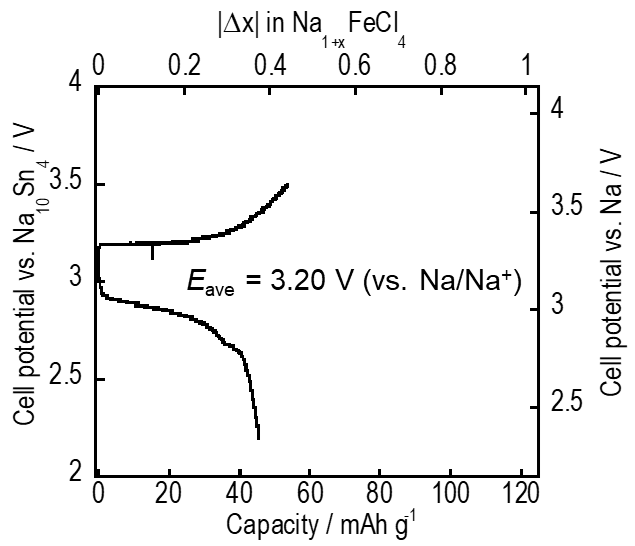
**

**Figure S3.** Constant-current charge–discharge curves of the all-solid-state sodium-ion battery (Na_10_Sn_4_+AB | Na_2.25_Y_0.25_Zr_0.75_Cl_6_ | NaFeCl_4_+KB) with NaFeCl_4_ electrode without electrolyte (NaFeCl_4_:KB = 90:10 wt.%) and chloride electrolyte Na_2.25_Y_0.25_Zr_0.75_Cl_6_.

**Supplementary Material 3.** **Characterization of NaFeCl_4_ electrode**

**Figure. S4.** d*Q*/d*V* curves of constant-current charge–discharge curves of the ASSB (Na_10_Sn_4_+AB | Na_3_PS_4_ | Na_2.25_Y_0.25_Zr_0.75_Cl_6_ | NaFeCl_4_+KB) using a NaFeCl_4_ electrode without electrolyte (NaFeCl_4_:KB = 90:10 wt.%) and with the bilayer electrolyte (Na_3_PS_4_ | Na_2.25_Y_0.25_Zr_0.75_Cl_6_).


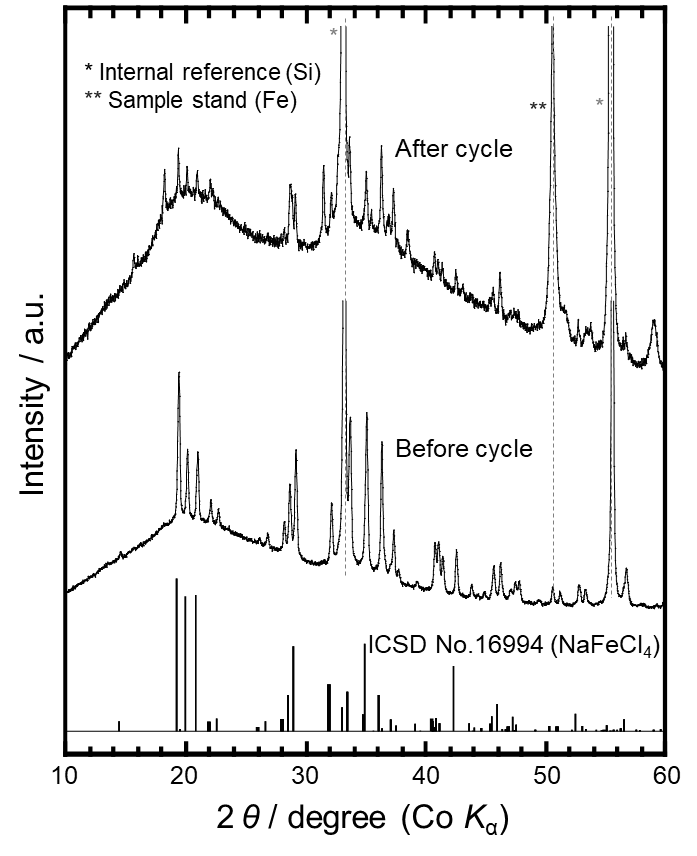
**Figure S5.** XRD patterns of the NaFeCl_4_ electrode before and after charging.
